# Supplementary material for: Frequency of Hepatitis B Virus Resistance Mutations in Women Using Tenofovir Gel as Pre-Exposure Prophylaxis
Source: Viruses. 2019 Jun 19;11(6):569. doi: 10.3390/v11060569 (PMC6630952; doi:10.3390/v11060569)
Supplement: Supplementary file 1 [file viruses-11-00569-s001.pdf]

Supplementary Table 1: Clinical and virological characteristics of the 37 women infected with HBV

PID	Age (Years)	Group assignment	HBeAg status	HBV DNA (copies/ml)*	ALT* (IU/L)	AST* (IU/L)	Any grade 2^ or higher liver related adverse event during follow up
120001	25	Tenofovir	Positive	5.61	48	50	yes
120018	20	Tenofovir	Negative	1.08	11	19	no
120078	18	Tenofovir	Negative	1.08	19	17	no
120176	24	Placebo	Negative	1.08	17	20	yes
120205	20	Placebo	Negative	0.00	39	30	yes
120253	37	Tenofovir	Negative	1.08	20	21	no
120260	29	Placebo	Negative	2.93	23	19	no
120314	37	Tenofovir	Negative	2.81	22	24	no
120323	27	Tenofovir	Negative	3.02	15	19	no
120325	30	Tenofovir	Negative	4.78	26	27	no
120338	22	Tenofovir	Negative	2.30	16	21	no
120466	26	Placebo	Negative	3.03	14	21	no
120489	20	Placebo	Negative	0.00	15	19	no
120499	18	Placebo	Negative	3.62	22	22	yes
120504	29	Tenofovir	Negative	2.63	22	24	no
145008	24	Tenofovir	Negative	2.59	24	25	no
145016	39	Placebo	Negative	$	18	18	no
200079	27	Placebo	Negative	1.56	31	31	no
200120	19	Placebo	Negative	2.67	22	22	no
200125	24	Tenofovir	Positive	0.00	20	36	no
200155	22	Tenofovir	Negative	1.95	22	26	no
200178	31	Placebo	Negative	1.67	15	18	no
200228	23	Tenofovir	Negative	3.16	10	25	no
200246	18	Tenofovir	Negative	2.16	31	25	no
200323	22	Placebo	Positive	0.00	12	15	yes
200342	24	Placebo	Negative	0.00	18	17	no
200355	32	Tenofovir	Negative	0.00	8	18	no
200368	18	Placebo	Positive	8.04	33	56	no
200371	30	Tenofovir	Negative	3.54	22	20	no
200427	31	Placebo	Negative	2.91	22	28	no
200468	26	Tenofovir	Negative	$	12#	17#	no
200489	22	Tenofovir	Negative	3.97	18	22	no
200515	19	Placebo	Negative	2.99	21	19	no
200533	24	Placebo	Negative	1.83	14	19	yes
200571	19	Tenofovir	Negative	2.72	18	26	no
200575	18	Tenofovir	Negative	3.21	19	23	yes
200582	21	Placebo	Negative	3.71	26	24	no

*results from study exit samples. $insufficient storage sample available for testing in 1 participant and 1 participant was lost to follow-up; ^grade 2 adverse event defined as >2.5 upper limit of normal; #results from study month 11
